# Supplementary material for: Are carbon emissions trading and green financial instruments synergistic? -Comprehensive quantitative research based on content analysis
Source: PLoS One. 2024 Mar 7;19(3):e0298601. doi: 10.1371/journal.pone.0298601 (PMC10919601; doi:10.1371/journal.pone.0298601)
Supplement: S1 Data — (ZIP) [file pone.0298601.s001.zip › Data/地级市Z_F工作报告.zip!/╔╜╬≈/╦╖╓▌╩╨/└┤╫╘╣1⁄2╓┌║┼í╛┬φ┐╦╩2╛▌═°í┐_2011.docx]

各位代表：

　　现在，我代表市人民政府向大会作工作报告，请予审议，并请市政协委员和其他列席人员提出意见。

　　一、“十一五”时期国民经济和社会发展回顾

　　“十一五”时期是我市发展史上极不平凡的五年。五年来，面对国际金融危机的严重冲击，面对经济结构深层次矛盾的重重困扰，面对保增长保民生保稳定的繁重任务，我们在省委、省政府和市委的坚强领导下，深入贯彻落实科学发展观，大力弘扬右玉精神，迎难而上，奋力进取，开拓创新，扎实工作，经济发展逆势而上，社会事业和各项工作都取得显著成就，夺取了“十一五”的全面胜利。“十一五”时期，是我市建市以来经济社会发展最快、效益最好、人民群众得到实惠最多的时期。

　　国民经济持续快速发展，综合经济实力迈上新台阶。全市地区生产总值达到670.15亿元，是“十五”末的3倍，年均增长14.6%，绝对额从全省的第11位前移至第9位；财政总收入达到132.5亿元，是“十五”末的3.8倍，年均增长30.5%，绝对额从全省的第10位前移至第8位；一般预算收入达到55.8亿元，是“十五”末的4.9倍，年均增长37.6%，绝对额从全省的第11位前移至第6位；工业增加值达到360亿元，年均增长24.5%；社会消费品零售总额达到139.6亿元，年均增长20.9%；城镇居民人均可支配收入达到17558元，农民人均纯收入达到5903元，均在“十五”末的基础上翻了一番。“十一五”期间，全社会固定资产投资累计完成1145.4亿元，是“十五”时期的4.7倍。经济总量的迅速扩大和效益的明显提高，说明我市的经济发展已经站在一个新的历史平台上。

　　大力推进产业结构调整，转型发展迈出较大步伐。制定实施了九大产业调整规划，启动实施了一大批调产项目，传统产业加快优化升级，新兴产业迅速发育成长。煤炭资源整合、煤矿兼并重组取得重大成果。煤炭产业的集约化、规模化、机械化和资源回收率、安全保障能力得到全面提高，煤炭工业进入了一个全新的发展阶段。205座地方煤矿整合为66座，矿井数量减少三分之二；平均单井生产规模由年产16万吨提高到129万吨，增加了7倍；保留矿井全部达到90万吨以上规模，全部实现机械化开采，资源回收率由20%左右提高到75%。全市原煤生产能力、洗选能力、发运能力均达到2亿吨。下决心推行了煤炭销售统一管理制度，煤炭销售秩序得到规范，取得明显经济效益。去年，全市煤炭产量达到1.6亿吨，位居全国前列；煤矿百万吨死亡率0.03，为全省全国先进水平。电力工业进一步发展壮大。大力发展煤矸石发电、热电联供、风电、太阳能光伏发电项目，五年新增电力装机容量109.45万千瓦，在建的各类发电项目全部投产后，全市电力装机容量将达到741.4万千瓦。非煤电产业快速发展。陶瓷、乳制品、建材、医药、化工等产业得到扩张。日用瓷生产能力达到11.5亿件，乳制品加工能力达到61万吨，干法水泥生产能力达到500万吨，合成氨生产能力达到15万吨。循环经济蓬勃发展。以煤电为基础，煤电冶、煤建材、煤化工等产业链条不断延伸壮大，资源综合利用率明显提高。中煤金海洋工业园区成为全省十大循环经济示范园区之一。服务业得到长足发展。现代物流业迅速扩张，机电设备维修业形成规模，旅游业日益兴旺，应县木塔和右玉县成为国家4A级旅游景区。招商引资成效突出，在历届“中博会”、“能博会”等大型招商活动中，我市签约项目数、拟引资额、总投资额多次位居全省第一。五年来，共有273个项目到位外来资金453.8亿元，为产业结构调整和经济发展增添了活力。

　　大力实施强农惠农政策，“三农”工作取得新成效。在认真落实中央和省各项强农惠农政策的同时，出台了扶持种粮大户、养殖园区、设施农业、玉米和甜菜种植等惠农政策。“十一五”期间，全市财政“三农”投入达33.9亿元，是“十五”时期的3.5倍。农田水利建设得到加强，完成节水灌溉农田、高标准旱作农田、中低产田改造100万亩，新修改造10座水库，水地面积达到180万亩，农业综合生产能力稳步提升。去年，粮食产量达到17.95亿斤，创历史新高。设施农业加快发展，去年新增日光温室和移动大棚4万亩。畜牧业保持稳定发展，全市建成奶牛养殖园区203个，存栏奶牛达到15.8万头，年出栏肉羊达到100万只。农业产业化水平得到提升，40家企业进入全省农产品加工龙头企业“513”工程。新农村建设扎实推进。520个试点村和重点推进村完成了新农村建设任务。特别是下大力气推进了农村“五个全覆盖”工程，两年投资20亿元，所有建制村实现了村通水泥路（油路）、中小学校舍安全改造、广播电视、村卫生室和饮水安全全覆盖。深入开展扶贫工作，1.1万农村贫困人口实现脱贫。经过五年的不懈奋斗，我市的特色农业、现代农业迅速壮大，农村面貌发生巨大变化，农民生活显著改善。

　　大力推进基础设施建设和生态建设，宜居城市建设迈上新水平。中心城市建设规模空前。按照“南改、北延、西拓、东扩”的总体思路，新建改建十余条主干道，实现了城市外环成型、路街贯通，城市建成区面积由7.8平方公里扩展到30平方公里，形成了158平方公里的城市大框架，为城市长远发展打下了良好基础。图书馆、博物馆、体育馆、文化艺术中心、火车站新站房、人民公园等一批重要公共设施投入使用，城市功能得到完善。大力实施绿化、亮化、净化工程，建成区绿化覆盖率达到44.82%，荣获“山西省园林城市”称号，城市形象得到提升。特色城镇建设取得新进展。右玉县被评为联合国最佳宜居生态县，怀仁县城被评为国家园林县城，朔城区老城改造被住建部评为中国十大最具影响力工程之一，应县和谐拆迁模式、平鲁区一城十镇百村模式受到中央和省领导的肯定。城镇的辐射带动作用进一步加强，城乡一体化进程不断加快。生态环境保护成效明显。大力推进造林绿化，坚持“挖一吨煤栽一棵树”，五年营造林165.4万亩，森林覆盖率由“十五”末的13.1%提高到18.02%。强力推进节能减排，二氧化硫和化学需氧量排放量、万元地区生产总值综合能耗均超额完成“十一五”任务，城市空气质量稳定提高。广大群众切身感受到，我们的城市越来越漂亮，越来越宜居，一个山青水秀、满目苍翠的美丽朔州已经崛起在塞外大地上！

　　更加注重保障和改善民生，社会事业加快发展。“十一五”时期，我们大幅度增加民生投入，全市财政在教育、卫生、就业、社会保障四大领域累计投入100.5亿元，是“十五”时期的4.7倍。教育事业全面提升。 “两免一补”政策全面落实。新改扩建农村寄宿制学校235所，实施了寄宿制学校营养餐工程和校车工程，我市在全国教育工作会议上介绍了发展义务教育的经验，平鲁区荣获全国义务教育均衡发展先进地区称号。全市兴建10所高中（新校区），高中阶段毛入学率大幅提升。朔州师专和职业技术学院建成使用，高等教育发展进入新阶段。医疗卫生事业取得较大突破。市中心医院、急救中心、疾控中心相继建成。县级医院建设全面加强，六县区县级医院新建了7座住院楼。乡镇卫生院得到全面改造，村卫生室实现了全覆盖。医药卫生体制改革全面推进，基层医疗卫生机构实行了基本药物制度。人口和计划生育工作在连续三年获得省政府综合先进奖的基础上，去年获得全省二类市综合考核第一名。就业形势保持稳定，五年新增城镇就业12.8万人。覆盖城乡的社会保障体系建设取得突破性进展。城镇居民基本医疗保险制度、新型农村合作医疗制度全面建立，群众看病难、看病贵问题得到有效缓解。最低生活保障制度实现全覆盖，城乡社会救助体系基本建立。新型农村社会养老保险试点工作稳步推进，并在全国首家推出65岁以上农村居民生活补贴制度。社会福利、优抚安置、慈善和残疾人事业都取得新进展。全市社会保障基金积累24.5亿元，比五年前增加了20亿元。保障性住房建设大规模推进，全市五年共投资90亿元，建设各类保障性住房73638套、612.77万平方米，其中市区拆迁棚户区300万平方米。还改造了4390户农村特困群众和农村贫困残疾人危房。我们正在进入一个人人享有社会保障的新时代！

　　科技、文化、体育等社会事业全面进步。地方财政科技投入逐年增长，科技研发取得了一系列重要成果，获得国内专利654项，国际专利2项，特别是粉煤灰综合利用研究成果全国领先。加强文化设施建设，推进文化体制改革，公共文化服务体系建设明显加快。朔州秧歌、晋北道情、梨花春酿造工艺成功申报国家非物质文化遗产保护项目。右卫镇和旧广武村被列为山西省历史文化名镇名村。城乡公共体育设施大幅度增加，群众性体育活动广泛开展。民族宗教、妇女儿童、地震气象等各项社会事业都取得了新成绩。

　　安全稳定呈现新局面。坚持不懈狠抓安全生产工作，建立健全了安全生产监管机制，强化了基层基础工作，安全生产形势明显好转。加快健全应急管理体系，在全国首家成立突发环境事件应急救援中心，受到环保部和公安部消防局的肯定。认真做好信访工作，加强社会治安综合治理，创新和加强社会管理，社会保持和谐稳定。积极支持国防后备力量建设和人防事业发展，国防教育基地、人防指挥所、防空疏散基地相继开工。同时，全力以赴支援四川汶川、青海玉树抗震救灾，支援南方地区抗击雨雪冰冻灾害，千方百计保障全国电煤供应，做出了朔州人民应有的贡献。

　　坚持依法行政，民主法制建设稳步推进。市政府自觉接受人大监督和政协的民主监督，积极支持各民主党派、工商联、人民团体和无党派人士参政议政。五年来共办理人大代表建议和政协委员提案1070件。坚持依法行政，不断加强政府法制建设，“五五”普法顺利完成。深入开展廉政建设，扎实推进煤焦领域反腐败斗争和工程建设领域突出问题治理。不断深化行政管理体制改革，加快转变政府职能，全面完成了新一轮政府机构改革，政府自身建设取得积极进展。

　　五年来，面对复杂严峻的宏观形势，特别是在国际金融危机的严重的冲击下，我市经济经得起考验，抗得住风险，主要经济指标增速在全省一直领先，多项重要工作走在全省前列，为全省应对金融危机、经济回升向好做出了突出贡献。在全省目标责任考核中，我市2008年名列全省第二，2009年和2010年连续两年夺得全省第一，连续两年被省委省政府表彰为优秀市。“十一五”时期综合考核，我市被评为全省经济社会发展优秀市。同时，我市还获得中国最具创新力城市、中国低碳贡献城市、中国经济转型示范城市等多项荣誉。五年来，全市人民在创造经济社会发展非凡成绩的同时，也创造了丰富的精神财富。特别是在艰苦奋斗中铸就升华的右玉精神，已经走出山西，走向全国，成为激励我们前进的精神动力。

　　各位代表，过去的五年，是我们在科学发展观指引下，攻坚克难、锐意进取、苦干实干的五年，也是经济社会又好又快发展，各项事业欣欣向荣，城乡面貌日新月异的五年。现在，我向大会郑重报告：我市“十一五”规划确定的目标任务已全部圆满完成！向人民群众承诺的各件实事已全部兑现！

　　回顾过去五年的历程，我们深切地感受到，之所以能够取得这样的好成绩，一是善于把握机遇，积极主动争取，打造了发展新优势。特别是在中央扩内需保增长政策出台之后，我们快速反应，主动出击，积极争资金争项目，取得了全省县均第一的好成绩，掀起了基础建设新高潮，不仅有效应对了金融危机的冲击，而且为长远发展打下了坚实基础。二是面对困难不退缩，沉着应对，因势利导，实现了化危为机。特别是在问题奶粉事件发生后，我们果断决策，迅速行动，采取了一系列有效措施，确保历经几十年艰苦努力培育而成的乳品业健康发展。三是始终坚持解放思想，先行先试，赢得了发展先机。特别是在煤矿兼并重组和机械化改造上，我们始终在全省走先一步，实现了煤炭工业正常生产和持续稳定发展，为全市经济“一路领先”、“一路飘红”发挥了重要作用。四是始终坚持改革开放，为经济社会大发展增添了动力与活力。特别是招商引资工作，一直保持全省领先，引进了大量资金项目，弥补了我们自身发展力量的不足，增强了全市经济发展的后劲与实力。五是始终坚持以人为本，把保障和改善民生作为一切工作的出发点和落脚点。我们拿出大量资金，着力加快各项社会事业发展，人民群众得到了更多实惠。六是始终坚持紧紧依靠广大干部群众，凝聚各方智慧和力量，有效调动各方面的积极性。五年来，全市人民热爱朔州、建设朔州的热情空前高涨，形成了推动发展的强大合力。

　　各位代表，过去五年，我们是一步一个脚印走过来的，我们完全有理由为取得的成就感到无比喜悦与自豪！五年的成绩来之不易，积累的经验弥足珍贵。这是省委、省政府和市委正确领导的结果，是市人大、市政协有力监督和鼎力支持的结果，是全市人民团结奋斗、辛勤劳动的结果。在这里，我代表市人民政府，向全市人民和所有关心支持朔州发展的各界人士，表示崇高的敬意和衷心的感谢！

　　在肯定成绩的同时，我们也清醒地认识到，我市经济社会发展还面临不少困难和问题。产业结构单一，倚重资源的增长方式没有改变，转型发展面临严峻挑战；经济总量仍然偏小，社会事业还欠发达，跨越发展任务十分艰巨；城镇化水平偏低，城市功能还不完善，承载能力、集聚功能和辐射带动能力不强；生态环境还比较差，城乡差距、功能差距和区域差距还比较大；部分干部破解发展中深层次矛盾的办法不多，抢抓机遇的胆气和先行先试的闯劲不够。对此，我们一定高度重视，采取有力措施，认真加以解决。

　　二、“十二五”时期经济社会发展的指导思想和主要任务

　　“十二五”是全面建设小康社会的关键时期，是深化改革开放、加快转变经济发展方式的攻坚时期，也是转型跨越发展的重要时期，特别是我省建设资源型经济转型综合配套改革试验区为我们提供了新机遇。我们一定要坚定信心，振奋精神，抓住机遇，乘势而上，实现新的跨越。

　　根据《中共朔州市委关于制定国民经济和社会发展第十二个五年规划的建议》，市政府编制了《国民经济和社会发展第十二个五年规划纲要（草案）》，明确提出“十二五”时期要高举中国特色社会主义伟大旗帜，以邓小平理论和“三个代表”重要思想为指导，以科学发展为主题，以转型发展为主线，以再造一个新朔州为目标，牢牢把握转型综改试验重大机遇，紧紧围绕建设自然生态现代宜居的幸福新城的战略定位，加快推进工业新型化、农业现代化、市域城镇化、城乡生态化、基本公共服务均等化、文化产业化，解放思想，开放创新，奋力进取，拼搏争先，努力打造新基地、新优势、新朔州。

　　“十二五”时期经济社会发展的主要目标是：经济快速健康发展，主要经济指标实现翻番，地区生产总值年均增长15%，达到1500亿元；财政总收入、一般预算收入分别达到300亿元和120亿元；全社会固定资产投资累计达到4000亿元。经济结构调整取得重大进展，需求结构、产业结构和要素投入结构更趋合理，城乡区域发展协调性明显增强，自主创新能力不断提高，节能减排取得明显成效，生态环境显著改善，安全生产形势稳定好转。城乡居民收入普遍较快增加，城镇居民人均可支配收入达到36000元，年均增长15.4%；农民人均纯收入达到12500元，年均增长16.2%。社会建设明显加强，覆盖城乡居民的基本公共服务均等化程度明显提高，体系基本完善；社会主义民主法制更加健全，文化事业和文化产业加快发展，社会更加和谐稳定。改革开放不断深化，重要领域和关键环节改革取得较大进展，资源型经济转型的体制机制不断完善，政府职能进一步转变，发展环境明显优化，对外开放的广度和深度持续拓展。

　　“十二五”时期，经济社会发展的主要任务是：

　　（一）全力推进“四化一体东部新区”建设，打造转型跨越发展的先行示范区。深入实施区域发展战略，进一步优化产业布局和空间结构。以大运高速公路和北同蒲铁路为轴线，怀仁、应县、山阴、城区四县区32个乡镇，工业布局相对集中，城镇化条件较为成熟，生态建设初具规模，农业基础相对优越，是我市经济发展的黄金带。要以产业项目为支撑，以农村发展、农业增效、农民增收为核心，坚持工业化、城镇化、生态化、农业现代化统筹考虑，同步推进，使东部区域率先发展、加快发展，成为引领和带动全市转型跨越发展的示范区。

　　（二）强力推进“四化”建设，构建转型跨越发展的战略基础。加快工业新型化，坚持以煤为基、多元发展，以循环经济为主要路径，以大项目、大企业、大园区为载体，以全国工业固废综合利用示范基地建设为抓手，以建设立体能源基地和新兴产业基地为目标，改造提升传统产业，培育壮大新兴产业，加快发展服务业，构建具有朔州特色的现代产业体系。到“十二五”末，全市煤炭生产能力达到2.5亿吨，电力装机容量达到1600万千瓦，同时，努力建设1000万吨粉煤灰综合利用基地、百亿立方米煤制天然气基地、百万吨级新材料基地，真正形成多联产、全循环、高端化的发展局面。加快农业现代化，以建设高产优质高效生态畜牧基地为目标，进一步加大强农惠农力度，加强基础设施建设，发展现代农业，加快建设社会主义新农村。实施水利“双百”工程，投资100亿元，建设100万亩连片灌区。实施“双五十万亩”工程，治理50万亩盐碱地，建设50万亩节水改造农田。实施“三个20”工程，设施农业面积达到20万亩，新增水浇地20万亩，粮食产能达到20亿斤。继续抓好奶牛和肉羊两大优势产业，推进规模健康养殖，奶牛存栏达到25万头，年出栏肉羊达到300万只。加快推进农业产业化，全市农副产品加工转化率达到60%。进一步深化新农村建设，高标准实施新的农村“五个全覆盖”工程。加快市域城镇化，以创建国家园林城市、全国文明城市、国家卫生城市、省环保模范城市、省双拥模范城市“五城联创”为目标，大力实施中心城市、县城、小城镇、新农村一体化发展战略，重点建设好一个中心城市，全面加强5个县城的特色城镇建设，规划建设好50个集镇、500个中心村。“十二五”末，全市城镇化率达到55%。扎实推进朔州机场等重点工程建设，提高城市的辐射带动能力。加快城乡生态化，坚持绿色发展，坚定不移实施生态立市战略，大力推进造林绿化、生态修复和环境整治，构建资源节约型、环境友好型社会。科学规划林业生态建设“两带、两区、二十园”的总体布局。“十二五”末，全市森林覆盖率达到24%以上，市区集中供热普及率达到90%以上，县城集中供热普及率达到80%以上。到那时，朔州的天更蓝，山更绿，水更清，空气更清新，一个具有“北欧风光、塞北风情”的新朔州一定会展现在世人面前！

　　（三）加快发展社会事业和改善民生，营造转型跨越发展的和谐环境。深入实施科教兴市和人才强市战略，大力发展科技、教育、文化、卫生、体育等各项社会事业，着力改善民生。坚持把扩大就业放在更加突出的位置，实施更加积极的就业政策，五年城镇新增就业15万人。加快完善社会保障体系，进一步提高保障水平，实现城乡基本养老、基本医疗保障制度全覆盖。加大保障性住房建设力度，“十二五”期间基本解决人均住房建筑面积13平方米以下的城市低收入家庭住房困难问题。进一步深化医药卫生体制改革，健全基本医疗卫生制度，完善覆盖城乡居民的公共卫生服务体系、医疗服务体系、医疗保障体系。坚持计划生育基本国策，稳定低生育水平。加快发展体育事业，积极开展形式多样的群众性体育活动。大力加强文化建设，加快推进文化产业化，积极实施文化基础设施全覆盖工程，大手笔制作一批彰显朔州特色、右玉精神的文化产品。加快发展旅游业，促进文化与旅游深度融合，带动服务业发展。创新社会管理体制机制，加强社会治安综合治理，强化安全生产工作。加强国防动员和人民防空建设，推进军民融合发展。发展社会主义民主政治，建设社会主义精神文明，不断推进法治政府建设，维护社会和谐稳定。

　　（四）深化改革开放，为推动转型跨越发展提供强大动力和活力。抓住我省建设转型综改试验区这一重大机遇，充分利用这一大品牌、大载体、大平台，紧紧围绕建设全省转型发展示范区、跨越发展领先区、循环经济集聚区和现代化工业新城，大力推动转型跨越发展。坚持解放思想，大胆探索，加强政策支撑，创新体制机制，力争在资金、土地、环境等要素配置上取得突破，在新建煤电项目与循环经济协调发展等方面作出探索。继续大力实施开放引进战略，强化服务，优化环境，不断提高对外开放水平。

　　各位代表，这些目标和任务体现了省委省政府的要求与朔州实际的结合，体现了长期目标与短期目标的衔接，体现了转型发展与跨越发展的统一，体现了打造新基地、拓展新优势、建设新朔州的要求。我们坚信，只要全市上下同心同德，团结拼搏，就一定能够谱写朔州转型跨越发展的新篇章！

　　三、2011年经济社会发展目标任务和工作重点

　　今年是中国共产党成立九十周年，是实施“十二五”规划的开局之年，是全面推动转型跨越发展的关键之年，也是我们打造新基地新优势新朔州、努力建设自然生态现代宜居幸福新城的首战之年。做好今年的工作，意义十分重大。

　　今年经济社会发展的主要预期目标是：地区生产总值达到780亿元，增长13 %；规模以上工业增加值达到430亿元，增长16%；全社会固定资产投资达到464亿元，增长24%；财政总收入达到155亿元，增长16.98%；一般预算收入达到65.7亿元，增长17.84%；社会消费品零售总额达到162亿元，增长16%；城镇居民人均可支配收入达到19313.5元，增长10%；农民人均纯收入达到6611元，增长12%；城镇新增就业岗位1.5万个，城镇登记失业率控制在4%以内，居民消费价格总水平涨幅控制在4%左右。

　　约束性指标是：万元地区生产总值综合能耗下降3.5%，万元地区生产总值二氧化碳排放量下降3.5%，二氧化硫、化学需氧量排放量分别下降1.5%和2%,氨氮、烟尘、粉尘排放量分别下降1.5%、2%、2%，万元工业增加值用水量下降5%。

　　今年，重点要抓好以下几方面工作：

　　（一）迅速启动“四化一体东部新区”建设，在全省率先走出一条转型跨越发展的新路子

　　“四化一体东部新区”，总面积4000平方公里，人口90万，城镇布局相对集中，规模以上工业企业占到全市的85%，农民人均纯收入平均达7600元，具有率先发展的基础。建设“四化一体东部新区”，就是要将其作为全面推进综改试验区和农村改革试验区建设的一个突破口和试验田，坚持以工业化带动城镇化、促进生态化、推动农业现代化，率先在全省走出一条“四化一体”推进转型跨越发展的新路子。要本着科学规划、快速起步，整合资源、集中投入，协调联动、形成合力的原则，迅速启动“四化一体东部新区”建设，力争一年见成效，两年大变样，三年创一流。到“十二五”末，区域内规模以上工业企业由现在的180家增加到230家，工业增加值由300亿元增加到700亿元；建成容纳5000人以上中心集镇20个，容纳1000人以上中心村200个，城镇化率达到70%；林木绿化覆盖率由20%提高到35%；农副产品加工企业达到200家，销售收入突破百亿元；农民人均纯收入提高到1.6万元，城镇居民人均可支配收入率先达到3.6万元，使4000平方公里的大地成为青山绿树分外妖娆，田园村镇交相辉映，经济社会繁荣兴旺，人民群众富裕幸福的乐园，成为带动全市转型跨越发展的龙头。

　　（二）以建设全省循环经济集聚区为重点，着力打造新型工业基地，加快工业新型化进程

　　要努力实施好“四大战略”：

　　大力实施传统产业优化提升战略，着力提高煤炭工业素质。以建设本质安全型现代化矿井为目标，全面推进煤矿机械化升级改造和安全生产标准化矿井建设，年内力争66座保留煤矿全部开工建设，20座矿井完成机械化改造并通过验收，16座矿井建成安全生产标准化矿井。着力打造大型龙头企业，支持中煤平朔“双千亿”工程和金海洋“双五百亿”工程加快实施，争取同煤麻家梁矿投产。全市煤炭产量力争达到2亿吨。

　　大力实施立体能源和新能源战略，加快发展电力工业。鼓励煤电一体化发展，实现煤电产业良性循环。进一步发展煤矸石发电、风力发电、太阳能发电、生物质能发电，拓展能源利用新领域，优化能源结构。加快建设神头一电厂2×60万千瓦和怀仁2×20万千瓦、右玉京玉2×30万千瓦、山阴昱光2×30万千瓦等3个矸石电厂、9个风电、2个光伏发电项目（总装机容量326万千瓦），力争华电2×30万千瓦热电联供项目开工建设。积极推进“十二五”规划确定的12个火电项目和21个风电项目的前期工作，争取早日开工。支持应县晶都和开发区中冶恩菲两个100万千伏光伏园区加快建设，发展新能源产业。加强电网建设，加快应县特高压输变电工程、平鲁500千伏输变电站等工程建设，进一步提高朔州的电力资源配置和输送能力。

　　大力实施多元发展战略，积极培育新兴产业。进一步加快陶瓷、医药、新型建材、装备制造等产业的发展步伐。以园区为载体，下力气抓好100个非煤电项目。支持平朔非煤工业园南非维艾西公司箱斗铲斗和大唐金凤风机组装、应县赛特1万台电梯等项目加快建设，发展装备制造业。支持平朔公司建设120万条轮胎、500万平方米运输胶带等项目，推动化工业拓展领域。支持美国康明斯公司发动机维修等项目落地建设，带动开发区进一步形成生产性服务业发展的集聚区。加快建设朔城区金圆、新时代、金海洋、怀仁同煤、右玉神固等新型干法水泥项目，形成1000万吨水泥生产能力，壮大建材业规模。以怀仁金沙滩陶瓷工业园区和应县陶瓷工业园区为重点，抓好恒源陶瓷生产研发基地、应县御锦800万平方米聚晶微粉抛光砖、明禾4000万件无铅骨瓷等项目，推动陶瓷业升级上档。以怀仁医药工业园区为重点，扶持诺成制药、同星土霉素等项目发展，壮大制药业规模。要把新兴产业特别是高新技术产业放在更加突出的位置，使新能源、新材料、新技术产业成为全市经济发展的新引擎。

　　大力实施绿色低碳发展战略，全力推进循环经济发展。坚持把循环经济作为改造提升传统产业的主要手段，作为新产业、新项目的准入门槛，作为资源配置的优先领域，推动资源循环式利用、产业循环化发展。要进一步加大对循环经济的政策支持和投入力度。认真编制工业固废综合利用示范基地建设的工作方案和产业发展规划，实施好循环经济发展意见，加快发展煤矸石、粉煤灰和电厂脱硫石膏等工业固废综合利用产业，促进循环产业链延伸壮大。煤炭企业要逐步实现30%的原煤产量就地转化，电力企业要全部消化粉煤灰和脱硫石膏，对矸石和粉煤灰制砖企业实行政府补贴。要加快建设循环经济工业园区。全面推进平朔、金海洋、右玉煤电、神头粉煤灰综合利用等循环经济工业园区的规划建设工作，努力将其打造成资源集约利用、产业集群发展、服务集聚配套的工业园区，成为转型跨越发展的标志性工程。要着力抓好重点项目。支持平朔公司20万吨粉煤灰提取氧化铝和白碳黑项目加快建设，推动山西国际电力集团和大唐公司两个40亿立方米煤制天然气项目早日开展实质性工作，支持金海洋公司利用粉煤灰提取100万吨硅铝铁合金、100万吨金属镁、50万吨镁合金、520万吨镁渣水泥一体化工程建设，支持润臻新技术公司利用粉煤灰、脱硫石膏生产3000万平方米纸面石膏板和150万平方米塑性复合板材项目尽快投产达效，带动全市循环经济实现大突破、工业新型化迈上新水平。今年要突出抓好神头粉煤灰综合利用工业园区，尽快完成基础设施建设，推动国能神州20万吨粉煤灰提取氧化铝和白碳黑、晋源新型建材公司60万立方米粉煤灰砌块等项目开工建设，力争把这个园区打造成全国一流的粉煤灰综合利用示范园区。

　　（三）坚持集约化规模化产业化方向，着力打造生态畜牧基地，加快农业现代化进程

　　努力做到“四个提高”：

　　以发展设施农业为重点，大力提高农业集约化水平。进一步优化种植结构，继续抓好优质玉米、马铃薯、小杂粮、蔬菜、胡麻、甜菜六大种植基地，重点抓好4万亩设施农业、 红旗牧场万亩现代农业综合示范园区和山阴万亩盐碱地改良示范区建设。加大科技兴农力度，实施项目强农战略。建立健全农业标准化体系，全面推进农产品产地认证，打造区域特色农业品牌。

　　以养殖园区建设为重点，大力提高畜牧业规模化发展水平。继续推进规模健康养殖，高标准建设一批整洁美观、技术配套、功能完善、管理科学的养殖园区，带动奶牛和肉羊两大优势产业快速壮大。年内新建奶牛小区50个、肉羊小区50个，奶牛存栏达到17.3万头，肉羊饲养量达到250万只，争取成为全省奶牛基地、肉羊基地和盐碱地改良示范基地三大工程建设的试点。在不折不扣落实好国家和省各项强农惠农政策的基础上，今年市财政将投入1亿元，实行灌区水价补贴、玉米地膜补贴、设施农业补贴、养殖园区补贴等十项惠农政策，促进农业增产、农民增收。

　　以龙头企业建设为重点，大力提高农业产业化经营水平。积极扶持古城、雅士利、中大科技等进入全省“513”工程的40家龙头企业做大做强，着力培育一批销售收入超10亿元和5亿元的龙头企业集群，推进农产品精深加工，提高农业附加值，力争农产品销售收入突破60亿元。加快农产品物流销售体系建设，年内新建改建山阴雁门关农产品物流大市场、朔城区大型农产品物流配送中心、怀仁南小寨肉羊和皮毛交易市场，改造提升应县南河种蔬菜批发市场，进一步提高农业市场化水平。

　　以新的“五个全覆盖”为重点，大力提高新农村建设水平。新增新农村建设重点推进村113个，总数达到633个村，超过全市农村总数的三分之一。要按照省委省政府的统一安排部署，全面启动实施农村新的“五个全覆盖”工程，即用两年时间，实现农村街巷硬化、农村便民连锁店、农村文化体育场所、农村职业教育免费、新型农村社会养老保险全覆盖，进一步改善农村经济社会发展条件，使广大农民群众过上更加美好的生活！

　　（四）坚持“中心城市、县城、小城镇、新农村”四位一体战略，着力打造自然生态现代宜居幸福新城，加快市域城镇化进程

　　城镇化是现代化的重要载体和基本推动力。要切实加大城镇化建设力度，实现以工补农、以城带乡、共同发展、整体腾飞。

　　要把基础设施建设作为推进市域城镇化的重要前提。继续加快中心城市建设。围绕城市让人民生活更美好的主题，坚持改造旧城区、拓展新市区、打造生态区、提升中心区的建设思路，进一步加大水源、供热、道路、绿化、亮化、净化六大工程建设力度，增强主城区辐射带动能力。年内要完成和平街、三水厂、污水处理厂再生水利用等续建工程，开工建设恢河大桥，改造市府东街、振武街。按照同城化的要求，高标准改造平朔一级路。加快神头二电厂、大唐电厂和双良公司热电联产集中供热项目建设进度，保证今冬为市民保质保量供好热。加大城市立体绿化力度，实施好平朔北路、张辽路、高速路出入口等道路绿化和民福西街立交桥景观绿化工程。切实抓好城中村改造，按照 “统一规划、市区联手、分年实施”的办法，力争用五年时间，全面完成城中村拆迁改造任务。全面启动“五城联创”工作，提高城市品位。积极推进以县城和中心乡镇为重点的小城镇建设。加快旧城改造步伐，大力推进城镇基础设施建设，发展城镇科教文化事业，完善城镇服务功能，增强城镇吸纳力和承载力。

　　要把产业发展作为推进市域城镇化的根本动力。大力发展城镇经济。中心城市和县城要加快发展各类服务业，特别是要抓好现代物流业和文化旅游业。现代物流业，要重点抓好市交通物流园区、城区居然之家、沃尔玛超市、豪德贸易广场二期、怀仁煤炭物流中心、华北皮革城、金沙滩物流园区、山阴贺庄煤炭物流中心、平鲁汽车城、右玉元堡煤炭物流中心。文化旅游业，要筹备建设市旅游服务中心，精心打造朔城区紫金山和神头海、平鲁区北固山和乌龙洞、山阴广武边塞文化旅游区和王家屏纪念园、怀仁金沙滩和清凉山、应县龙首山、右玉西口古道和古村古堡等一批景区景点。今年要集中力量推进应县木塔申报世界文化遗产工作，努力取得较大进展。大力发展县域经济。按照“一县一业、一村一品”的思路，明确发展方向，选准主导产业，强化政策保障，全力打造山阴奶牛、怀仁肉羊、右玉小杂粮、应县蔬菜、朔城区玉米、平鲁区马铃薯六个基地县和600个专业村，以农业现代化为基础、工业化为支撑、城镇化为载体，推动县域经济快速健康发展。要严格县域经济发展责任考评，促进各项工作落实。

　　要把制度创新作为推进市域城镇化的重要保证。以建设农村改革试验区为抓手，加快城乡空间布局、产业发展、基础设施和公共服务、就业和社会保障、社会管理一体化进程。积极推进户籍制度改革，放宽农民进城落户条件，凡是在城镇有固定住所和较稳定职业的，允许办为城镇户口。积极探索养老保险、医疗保险、保障性住房分配制度改革，使那些在城镇新落户的人员与原城镇居民享受同等待遇，特别是要解决好农民工子女入学等切身利益问题。

　　（五）坚持生态立市，着力打造“绿色朔州”，加快城乡生态化进程

　　强力推进造林绿化。按照“十二五”林业生态建设“两带、两区、二十园”的总体规划，今年营造林33万亩，重点抓好十大规模造林工程，即朔城区3万亩西山环城绿化工程、朔城区城西2万亩苗木基地建设工程、平鲁卧龙山5万亩绿化工程、平鲁太平山2万亩绿化工程、山阴广武1万亩绿化工程、山阴西山2万亩绿化工程、怀仁金沙滩5.6万亩二期绿化工程、应县53公里高速公路通道绿化工程、右玉42公里大呼高速公路通道绿化工程、右玉七彩山1.8万亩荒山造林工程。要以高水平的生态建设成果迎接全国“三北”防护林现场会和全国突出环境事件应急救援现场会在我市召开。

　　强力推进治污减排。努力减少二氧化碳排放，严格控制二氧化硫、化学需氧量、烟尘、粉尘等主要污染物排放。继续加大重点用煤行业的脱硫力度，启动火电行业脱硝工作。坚决拆除市区燃煤小锅炉，减少空气粉尘污染。完成平鲁、山阴、怀仁集中供热改造工程，加快平鲁、山阴、应县生活垃圾处理厂建设。

　　强力推进节能降耗。严格产业准入门槛，加强新上项目能耗评审工作，加快淘汰落后产能。落实政府优先和强制采购节能产品制度，降低能耗水平。政府投资项目要全部采用煤矸石或粉煤灰加工制造的节能环保砖，要在全市范围内逐步取消实心粘土砖。积极倡导绿色消费，扩大可再生能源应用，推进全社会节能。

　　（六）坚持不懈推进重点工程建设，夯实经济社会发展基础，增强发展后劲

　　坚持基础设施建设与产业开发并重、国有投资与民间投资并举的方针，进一步加强重点工程建设。

　　今年全市要启动实施208项重点工程，总投资1934亿元左右，当年计划完成投资450.5亿元。道路交通方面，实施“53522”建设工程，即配合好北同蒲增四线工程、大西高速客运专线、准朔铁路、大准至朔黄铁路连接线朔州段、准朔铁路朔山连接线等五项铁路工程的建设，扎实推进荣乌高速山阴至平鲁段、平朔高速公路、西纵高速朔州段三条高速公路的建设，完成虎山线改造、大石线应县县城改线、大忻线山阴县城改线、109国道右玉县城改线、大呼高速公路右玉连接线等五条干线公路改造工程，建设怀仁金沙滩至鹅毛口、朔城区朔广线至紫金山两条旅游公路和神电运煤通道二期工程、歇马关至西神头两条运煤通道。水利建设方面，开工建设西山引水、七里河改造、桑干河干渠建设、神头泉改造、太平窑二库五大工程；加快引黄北干工程建设，抓好大梁水库、尚希庄水库、海子湾水库、东石湖水库建设，特别要抓好神头、平鲁、山阴、怀仁四个水厂建设，确保10月1日前用上黄河水。城市建设方面，主要是朔城区老城改造、城中村改造和保障性住房建设。产业结构调整方面，要以升级改造为方向，以科技进步为支撑，加大投入，煤炭行业投资578亿元，电力行业投资505亿元，煤化工行业投资452亿元，高新技术产业投资36.2亿元。同时要抓好一批教育、卫生等社会事业发展的重点项目，总投资43亿元。

　　要创新投融资机制，为重点工程建设提供可靠的资金保障。积极引进交通、招商、晋商等银行在我市设立分支机构，加快发展村镇银行和小额贷款公司，健全中小企业政策性担保和融资性担保体系。鼓励龙头骨干企业上市直接融资，开辟融资新渠道。政府投资要重点放在强化公共服务和加强薄弱环节上，发挥好导向作用。

　　今年重点工程建设的投资规模，超过以往任何一年，任务极其艰巨。我们一定要集中精力抓项目，全力以赴搞建设，再掀重点工程建设新高潮，再打一场重点工程建设的大胜仗!

　　（七）以保障和改善民生为重点，加快各项社会事业发展，促进社会和谐稳定

　　优先发展教育事业。认真落实国务院三年行动计划，推动学前教育快速发展，在开发区、开发北延路经济适用房小区、民福立交桥南和小村旧城改造区四个地方各规划新建1所幼儿园，各县区要在县城内建设2—3所公立幼儿园。要以城市中小学布局调整和农村寄宿制学校建设为重点，促进教育均衡发展。加大职业高中和职业培训中心建设力度，启动朔州技校搬迁工作，促进职业教育快速发展。进一步完善师专各项配套设施建设，努力提升教学质量，争取年内通过教育部验收。积极推进山西能源学院建设，力争年内主体工程完工。

　　加快发展医疗卫生事业。深化医药卫生体制改革，基层医疗机构全面实行基本药物制度。加强医疗卫生信息化建设，建设1个市级医疗卫生信息中心，1个市级、6个县级、12个乡级远程医疗会诊中心。继续推进县乡村三级医疗机构达标工作，完成23个乡镇卫生院、502个村卫生室的升级改造，力争乡村两级医疗机构达标率达到95%以上。争取上级支持建设市全科医师培训基地，积极筹建市中医康复医院。加强食品药品检验能力建设，确保食品药品质量安全。从今年开始，我市将在全省率先开展每年对65岁以上老年人免费健康体检，让全市12.4万老年人享受公共财政的阳光。

　　加快推进科技文化体育事业。大力推进科技创新。筹建高新技术创业园区，创办专业技术研究院（所）。发展10家市级高新技术企业，扶持10家企业建立市级科技研发中心。实施“双百科技人才培育工程”，引进100名高层次人才，培养100名本地高级专业技术人才。繁荣发展文化体育事业。进一步完善城市文化基础设施，继续抓好乡镇综合文化站和农家书屋建设工作，延伸村村通广播电视工程，健全公共文化服务体系。加强文物保护，制定全市非物质文化遗产保护专项规划。加快城乡文化体育场馆建设，广泛开展群众性体育活动和全民健身活动，积极推进竞技体育发展。加快档案事业发展，开工建设市档案馆。

　　稳定和扩大就业。继续执行促进高校毕业生就业等扶持政策，切实解决好高校学生就业问题。大力发展劳动密集型产业，建立健全公共投资带动就业增长的机制，加强就业和再就业培训，努力扩大就业。

　　加快完善社会保障体系。稳步推进新型农村养老保险制度，增加朔城区和平鲁区两个试点。扎实推进城镇居民医疗保险制度，进一步扩大覆盖面。做好全省保障性住房建设试点工作，年内开工建设保障性住房47842套、508.89万平方米，完成投资31.2亿元。建设市儿童福利院、市社会福利院和市残疾人康复中心，促进民政和残疾人事业发展。

　　全面加强社会管理。坚持不懈抓好安全生产，深入贯彻落实国务院和省政府关于进一步加强企业安全生产工作的一系列要求，强化两个主体责任，进一步抓好以煤矿为重点的各行业各领域的安全生产工作，推动全市安全生产形势由明显好转向稳定好转迈进。加强防震减灾设施建设，完善突发事件应急处置机制。认真做好信访工作，正确处理各类社会矛盾，坚决纠正损害群众利益的突出问题。加强社区建设，提高社会文明程度，为城乡居民创造更好的生活条件。加强社会治安综合治理，严厉打击各类违法犯罪特别是严重暴力犯罪活动和黑恶势力，确保一方平安。

　　各位代表，不断提高人民群众的生活水平是我们一切工作的出发点和落脚点，我们将进一步加大投入力度，努力让全体人民学有所教、病有所医、老有所养、住有所居，让广大人民群众享受到更多的改革发展成果，生活得更加幸福、更有尊严！

　　（八）坚持先行先试，深化改革开放，争做转型综改试验区建设的排头兵

　　坚持以转型综改试验区建设为统揽，全面推进综合配套改革。加强组织领导，抓好综改试验区的启动实施。市里要成立领导组和办公室，统一领导、组织、协调改革试验工作，形成协调有序、推动有力的工作机制。要抓紧编制并实施好总体方案和财税、金融、投资、土地资源、生态环境等各个专项方案，指导改革有序推进。要认真实施好各个试验试点项目。我市已经被国家和省里确定了一批改革试点项目。怀仁县和右玉县被列为国家可持续发展试验区，还有工信部工业固废综合利用示范基地、商务部综合执法试点，农业部农村改革试验区正在争取之中。省级项目有民政事业综合改革示范区、统计综合改革合作示范区、医疗卫生信息化试点等，应县被列为省级可持续发展试验区，市经济开发区被列为省级资源环境可持续发展综合改革试验区。我们要在上级部门的指导下，扎扎实实把各项工作稳步推进，发挥好改革创新的示范带动作用，并争取更多的改革试验田放在朔州。

　　坚持大开放促进大发展，进一步加大招商引资力度。要以“中博会”、“能博会”等大型招商引资活动为平台，创新招商方式，拓展引资渠道，吸引更多的人才、资金、项目、技术落户朔州。重点吸引国内外大企业、重点民营企业来朔投资兴业，为朔州建设服务。今年，全市力争落地招商引资项目150个，实际到位资金210亿元以上。

　　（九）加强政府自身建设，为转型跨越发展提供坚强保障

　　推进转型跨越发展，对政府自身建设提出了更高的要求。我们一定要坚持执政为民的理念，以改革创新的精神，全面履行职能，狠抓工作落实，不断提高行政能力，努力建设人民满意的政府。

　　转变政府职能，提高行政效能。要进一步深化行政审批制度改革，精简审批事项，减少审批环节，转变政府职能。要切实增强服务意识和责任意识，坚决克服形式主义、官僚主义，全面提高行政效能。

　　增强法治意识，推进依法行政。各级政府要自觉接受人大及其常委会监督，主动加强与政协联系，及时办理各类建议和提案。严格依照法定权限和程序行使权力，履行职责。坚持科学民主决策，完善重大事项公众参与、专家咨询论证和政府集体决策相结合的决策制度。加强政府法制工作，强化行政监督，规范行政行为，严格行政问责，提高政府的公信力和执行力。

　　发扬右玉精神，加强作风建设。每个政府工作人员都要始终保持良好的精神状态，对群众有感情，对工作有热情，对事业有激情，以高度的责任感和使命感，扎实做好各项工作。坚持一切为了群众，把实现好、发展好、维护好广大人民群众的根本利益作为工作的出发点和落脚点。坚持一切从实际出发，树立正确的政绩观，求真务实，真抓实干，创造出经得起实践、历史和人民群众检验的政绩。

　　坚持廉洁自律，做到为民务实清廉。各级政府要严格执行廉政准则，认真落实党风廉政建设责任制，对干部严格要求、严格教育、严格管理、严格监督，加强惩治和预防腐败体系建设。继续深入推进煤焦领域反腐败专项斗争和工程建设领域突出问题治理，认真开展重点领域渎职侵权问题专项整治工作，深化政务公开，确保公共权力在阳光下运行。大兴勤俭节约、艰苦朴素之风，坚决反对奢侈挥霍、铺张浪费，把有限的资金更多地用在发展经济、改善民生上。

　　各位代表，宏伟蓝图催人奋进，转型跨越时不我待。我们告别了辛勤耕耘、硕果累累的“十一五”，迎来了充满希望、大有可为的“十二五”。让我们在中共朔州市委的坚强领导下，解放思想，开拓创新，扎实工作，奋力争先，为打造新基地、新优势、新朔州和建设自然生态现代宜居的幸福新城而努力奋斗！
